# Special Section Guest Editorial: Photodynamic Therapy

**DOI:** 10.1117/1.JBO.25.6.063801

**Published:** 2020-05-01

**Authors:** Kimberley S. Samkoe, Jonathan P. Celli, Timothy C. Zhu

**Affiliations:** a Dartmouth College, Geisel School of Medicine and Thayer School of Engineering, Hanover, New Hampshire, United States; b University of Massachusetts Boston, Department of Physics, Boston, Massachusetts, United States; c Hospital of the University of Pennsylvania, Perelman School of Medicine, Department of Radiation Oncology, Philadelphia, Pennsylvania, United States

## Abstract

Guest Editors introduce the Special Section on Photodynamic Therapy for the Journal of Biomedical Optics, Volume 25, Issue 6.

This Special Section of Journal of Biomedical Optics Volume 25, Issue 6 is dedicated to Photodynamic Therapy (PDT), based in part on contributions from the 17^th^ biennial International Photodynamic Association World Congress held in Boston, MA. We are pleased to present a total of 12 papers demonstrating the latest and greatest developments in the field of PDT.

A key challenge in clinical PDT for any given disease application is the development and validation of tools, techniques and models for reliable dosimetry. Meaningful predictive modeling, measurement and monitoring of the dose deposited for any given treatment protocol can be particularly challenging for PDT. There are inherently a large number of parameters that govern localization of the photosensitizer, delivery of light, generation of cytotoxic photochemical products and ultimately the extent to which photoproducts are quenched by the target tissue. As such it is fitting that more than half of the papers submitted in this Special Issue emphasize various aspects of PDT dosimetry. Two papers focus on the use of multispectral detection for real-time dosimetry: paper 063810, by Moritz et al., focuses on the continuous, direct measurement of both singlet oxygen (SO) and photosensitizer luminescence during therapy using multispectral detection, while paper 063812, by Mousavi et al., demonstrates a multiexcitation and multiemission system for monitoring both fluorescence and reflectance to assess photosensitizer concentration and tissue optical properties. Scholzet al.10.1117/1.JBO.25.6.063806 (paper 063806) explores an innovative method of time-gated delayed fluorescence as an indirect measure of SO production. Izumotoet al.10.1117/1.JBO.25.6.063803 (paper 03803) and Shenget al.10.1117/1.JBO.25.6.063805 (paper 063805) explore *in silico* methods of predicting SO concentrations using explicit measurements of therapeutic parameters in 5-aminolevulinic acid glioma therapy and benzoporphyrin derivative monoacid ring A vascular therapy, respectively. Of note is utilization of fluorescence dosimetry based on smartphone camera for PDT implicit dosimetry (paper 063802, by Ruiz et al., and paper 063813, by Khan et al.) that can populate the use of PDT dosimetry in clinical setting. Judging from these exciting papers, there has been significant progress made in dosimetry of PDT.

In addition to this lineup of PDT dosimetry reports, this issue also features five papers introducing new techniques and technologies for enhancement of PDT and/or enabling new PDT-based applications. These studies include a comparison of single and dual-wavelength PDT treatment (paper 063804, by Kurakina et al.), the effect of Catechin on reducing the phototoxic effects of protoporphyrin IX (PpIX) mediated PDT (paper 063807, by Joniová and Wagnières), dual use dyes for both PDT and as photoacoustic image contrast (paper 063808, by Petrovic et al.), increasing the diagnostic depth of PpIX-based photodynamic diagnosis by using green excitation light (paper 063809, by Ihara et al.), and a laboratory-based LED array to enable streamlined preclinical PDT research (paper 063811, by Kercher et al.).

In closing, significant progress has been made in advancing PDT dosimetry, and bringing cost-effective PDT delivery and dosimetry tools in global health settings or more broadly for practicality and convenience. Innovative imaging and detection tools are being implemented in order to maximize therapeutic efficacy and improve overall patient outcomes. Overall, this special issue highlights the wide-ranging and exciting developments in PDT for treatment of human disease.

